# *Plasmodium knowlesi:* a superb *in vivo* nonhuman primate model of antigenic variation in malaria

**DOI:** 10.1017/S0031182017001135

**Published:** 2017-07-17

**Authors:** M. R. GALINSKI, S. A. LAPP, M. S. PETERSON, F. AY, C. J. JOYNER, K. G. LE ROCH, L. L. FONSECA, E. O. VOIT, THE MAHPIC CONSORTIUM

**Affiliations:** 1 Emory Vaccine Center, Yerkes National Primate Research Center, Emory University, Atlanta, GA, USA; 2 Division of Infectious Diseases, Department of Medicine, Emory University, Atlanta, GA, USA; 3 La Jolla Institute for Allergy and Immunology, La Jolla, CA 92037, USA; 4 Department of Cell Biology & Neuroscience, Center for Disease and Vector Research, Institute for Integrative Genome Biology, University of California Riverside, CA 92521, USA; 5 The Wallace H. Coulter Department of Biomedical Engineering, Georgia Institute of Technology and Emory University, Atlanta, Georgia, 30332-2000, USA; 6 Malaria Host–Pathogen Interaction Center, www.systemsbiology.emory.edu

**Keywords:** *SICAvar* genes, *var* genes, *Plasmodium falciparum*, epigenetics, mathematical models, systems biology, multi-omics, host–pathogen interactions, longitudinal infections, macaques

## Abstract

Antigenic variation in malaria was discovered in *Plasmodium knowlesi* studies involving longitudinal infections of rhesus macaques (*M. mulatta*). The variant proteins, known as the *P. knowlesi* Schizont Infected Cell Agglutination (SICA) antigens and the *P. falciparum* Erythrocyte Membrane Protein 1 (PfEMP1) antigens, expressed by the *SICAvar* and *var* multigene families, respectively, have been studied for over 30 years. Expression of the SICA antigens in *P. knowlesi* requires a splenic component, and specific antibodies are necessary for variant antigen switch events *in vivo*. Outstanding questions revolve around the role of the spleen and the mechanisms by which the expression of these variant antigen families are regulated. Importantly, the longitudinal dynamics and molecular mechanisms that govern variant antigen expression can be studied with *P. knowlesi* infection of its mammalian and vector hosts. Synchronous infections can be initiated with established clones and studied at multi-omic levels, with the benefit of computational tools from systems biology that permit the integration of datasets and the design of explanatory, predictive mathematical models. Here we provide an historical account of this topic, while highlighting the potential for maximizing the use of *P. knowlesi* – macaque model systems and summarizing exciting new progress in this area of research.

## INTRODUCTION

Antigenic variation of parasite-encoded proteins expressed at the surface of *Plasmodium* infected RBCs (iRBCs) is critical to parasite survival. In turn, host immune responses against these proteins are important for host survival and the establishment and maintenance of chronic asymptomatic malaria infections (Howard, 1984; Galinski and Corredor, 2004; Arnot and Jensen, 2011). Upon repeated exposure to parasites with different variant antigen repertoires, individuals are less likely to exhibit severe clinical complications and will eventually cease having symptomatic infections, despite being parasitemic (Bull *et al.* 1998). On a population level, antigenic variation has explained observations of reduced incidence of severe disease in older individuals living in high transmission settings. Importantly, aside from being the basis, by definition, of an unhealthy state, chronic parasitemias may aid the propagation of the parasite *via* transmission (Zhou *et al.* 2016). Taking all these aspects into account, understanding antigenic variation mechanisms and chronicity may lead to new strategies to boost the host immune system for clearing parasitemias and in turn support malaria elimination strategies (Alonso *et al.* 2011).

Malaria antigenic variation has been most prominently associated with the *Plasmodium falciparum* Erythrocyte Membrane Protein-1 (EMP-1) variant antigens, which are encoded by the large *var* multigene family with about 60 members dispersed throughout all 14 chromosomes, with the majority located near the telomeres (Leech *et al.* 1984; Baruch *et al.* 1995; Smith *et al.* 1995; Su *et al.* 1995). However, the phenomenon of antigenic variation was discovered in *Plasmodium knowlesi* (Brown and Brown, 1965). In *P. knowlesi*, the Schizont Infected Cell Agglutination (SICA) variant antigens (Howard *et al.* 1983) are encoded by the related large *SICAvar* multigene family, which has at least 136 members that are likewise dispersed on all 14 chromosomes, yet more evenly distributed than the *P. falciparum var* genes and without as much concentration near the telomeres (Al-Khedery *et al.* 1999; Corredor *et al.* 2004; Pain *et al.* 2008; Lapp *et al.* 2017). The initial groundbreaking studies from 1965 involved experimental longitudinal infections of *P. knowlesi* in rhesus macaques, and this work raised a number of critical questions regarding the mechanisms of antigenic variation that remain unanswered today. In contrast to *P. falciparum* and *P. knowlesi*, the *var* gene family is not present in the human malaria species *Plasmodium vivax*, *Plasmodium malariae* and *Plasmodium ovale* (Carlton *et al.* 2008; Rutledge *et al.* 2017). Since *P. knowlesi* was recognized as a zoonotic parasite and a widespread public health threat in South East Asia (Singh *et al.* 2004; Cox-Singh and Singh, 2008; Cox-Singh *et al.* 2008), the relative importance of understanding antigenic variation in this species has been escalating.

This paper puts forth the overarching hypothesis that molecular mechanisms of *Plasmodium* antigenic variation *in vivo,* regulated by specific host responses and factors, can be discovered or computationally inferred with modern methods of systems biology and possibly result in groundbreaking strategies for treating malaria infections. Given the widespread implications of antigenic variation in malaria, with hundreds of millions of cases estimated annually in about 100 countries, we believe this research area should gain increased traction with translational goals in mind.

## PERSPECTIVES

Importantly, the dynamics and molecular mechanisms of variant antigens in *P. knowlesi* can be studied *in vivo* using macaques, with highly synchronous blood-stage infections initiated by established (or new) SICA[+] and SICA[−] clones, which do or do not express the variant antigens, respectively (Barnwell *et al.* 1983*a*). The *P. knowlesi* rhesus macaque model system is the most advanced option for studying the regulatory mechanisms of antigenic variation based on *in vivo* investigations, with the use of *in vivo* derived, well-characterized parasite clones. Moreover, this nonhuman primate (NHP) model allows for experimental passage through the mosquito host, thereby offering the opportunity to observe possible reset patterns of *SICAvar* gene expression and to monitor the within-host expression dynamics for the first time during the course of longitudinal infections. In addition, the *Anopheles dirus* mosquito can be reared in the laboratory, providing an experimental model system for the study of regulatory mechanisms of antigenic variation that may occur in the vector. This vector has been implicated in natural *P. knowlesi* and *P. falciparum* transmission in the region (Nakazawa *et al.* 2009; Marchand *et al.* 2011). The value of investigations of the related simian species *Plasmodium coatneyi* and *Plasmodium fragile,* which also undergo antigenic variation and share biological features akin to *P. falciparum*, should also be stressed (Handunnetti *et al.* 1987; Galinski and Corredor, 2004; Chien *et al.* 2016). In-depth comparative investigations of these species may be worth pursuing in the future, as would be studies of *P. knowlesi* in the natural macaque hosts from South East Asia, *Macaca fascicularis* and *Macaca nemestrina* (Cox-Singh *et al.* 2008; Divis *et al.* 2015; Maeno *et al.* 2015). It has been predicted that the human and simian malaria species share regulatory mechanisms, and it is likely that similar molecular and immunobiological host factors govern their expression (Barnwell *et al.* 1983*b*; Howard, 1984; Howard and Barnwell, 1984*b*; Korir and Galinski, 2006; Arnot and Jensen, 2011).

Model systems and advanced technologies have reached a level of sophistication allowing for the realistic characterization of the *in vivo* dynamics of *Plasmodium* antigenic variation, as well as the identification of specific *in vivo* host-parasite factors that regulate antigenic variation. This can be done by analyzing infected host and parasite blood samples from longitudinal infections and conducting high-throughput studies with multi-omic analyses (e.g., genomics, epigenomics, transcriptomics, proteomics, lipidomics, immunomics and metabolomics). A refined *Macaca mulatta* genome (Zimin *et al.* 2014) (Version 7.8) has been available, but a refined *P. knowlesi* genome (Pain *et al.* 2008) assembly with fully annotated *SICAvar* sequences has been lacking. To fill this need, over the past year, a high-quality *P. knowlesi de novo* genome sequence has been generated in the Malaria Host–Pathogen Interaction Center (MaHPIC) by combining long-read sequences (PacBio) and genome-wide high-throughput chromosome conformation capture (Hi-C) data to produce accurate, chromosome-level scaffolds, followed by automated and manual annotation, including for all *SICAvar* genes. This genome sequence has been named the ‘MaHPIC Pk Genome Sequence’ and is being reported elsewhere in this Special Issue (Lapp *et al.* 2017). This sequence, combined with sophisticated mathematical and computational analyses to determine how the *SICAvar* expression is affected by passage through the *Anopheles* vector and NHP hosts, will aid future research toward understanding the molecular factors regulating antigenic variation *in vivo* throughout the parasite's life cycle.

## HISTORICAL OVERVIEW

### The P. knowlesi–rhesus macaque model system: discovery of malaria antigenic variation and a regulatory role of the spleen

**Fig. 1.**
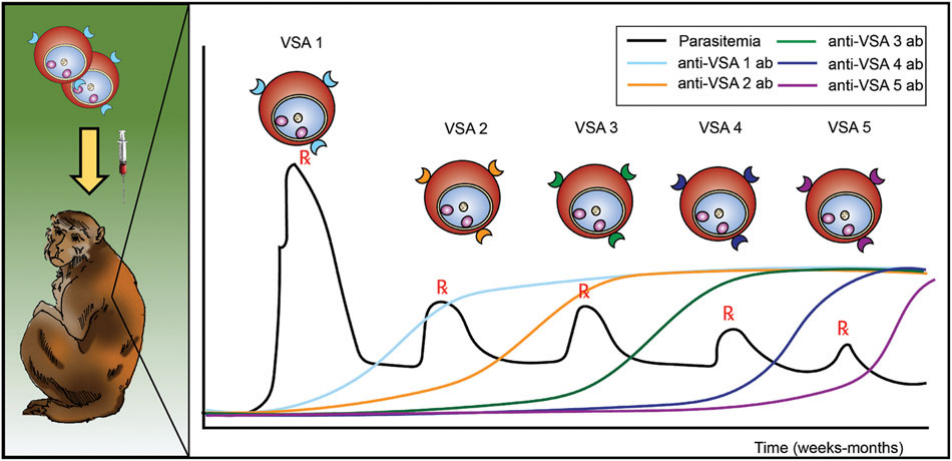
Schematic representing the longitudinal infection experiments performed with *P. knowlesi* in rhesus monkeys, demonstrating the phenomenon of malaria antigenic variation, and reported by K.N. Brown and I.N. Brown in 1965, in Nature (Brown and Brown, 1965). Different VSAs are expressed in the course of an infection, as antigenic variation occurs in response to the appearance of anti-VSA antibody (ab). VSA, variable surface antigens.

Antigenic variation in malaria was discovered in longitudinal experiments by inoculation of rhesus macaques with *P. knowlesi* blood-stage parasites (Brown and Brown, 1965). These studies used SICA agglutination assays (Eaton, 1938) to demonstrate that variant antigens were exposed at the surface of iRBCs and that the antigenicity changed during the course of an infection. The antigens were subsequently determined to be parasite-encoded proteins (Howard *et al.* 1983), and are often referred to, across species, as variable surface antigens or VSAs (Fig. 1). Immediately following this demonstration, efforts continued to understand the host responses to these antigens and to assess whether the changing of exposed antigens (alternatively referred to as ‘switching’) was a result of antibody induction or selection; the early studies concluded that both could be in play (Brown, 1973; Brown and Hills, 1974). At the time, it was unknown whether the host proteins were simply changing or if the parasite was producing different proteins at the surfaces of the host cells.

In 1983, nearly 20 years later, *in vivo* derived parasite clones were developed by Barnwell, Howard and others and shown to switch variant types in animals in the presence of specific antibodies, whereas the clones did not switch phenotypes in naïve animals (Barnwell *et al.* 1983*a*). The original and other *P. knowlesi* related clones were developed by micromanipulation of single schizonts and expansion in naïve rhesus, followed by cryopreservation as ring-stage forms (Barnwell *et al.* 1983*a*, and unpublished data confirming additional switched clones). As one example, the Pk1(A+) clone switched when inoculated into a rhesus that had been previously inoculated with Pk1(A+) parasites, and the Pk1(B+)1+ clone was derived from the resulting switched population. The Pk1(A+) and Pk1(B+)1+ clones express dominant SICA proteins that were characterized as doublets of 190 and 210 kDa, and 200 and 205 kDa, respectively (Howard *et al.* 1983). These were identified with radioiodination surface labeling and immunoprecipitation studies based on detergent extracts and variant specific polyclonal sera, to be expressed at the surface of the iRBCs, predictably with a transmembrane domain and internal cytoplasmic domain (Howard *et al.* 1983; Howard and Barnwell, 1984*a*, *b*, *c*, 1985; Howard *et al.* 1984). These studies provided confirmatory data to support the theory that the parasites were expressing distinct parasite-encoded antigens that changed with each antigenic switch. Comparable biochemical methods used to identify the SICA proteins were subsequently applied by Leech and others to identify EMP-1 in *P. falciparum.* They showed that the proteins from both species shared basic characteristics and, in particular, basic common regulatory mechanisms (Leech *et al.* 1984).

**Fig. 2.**
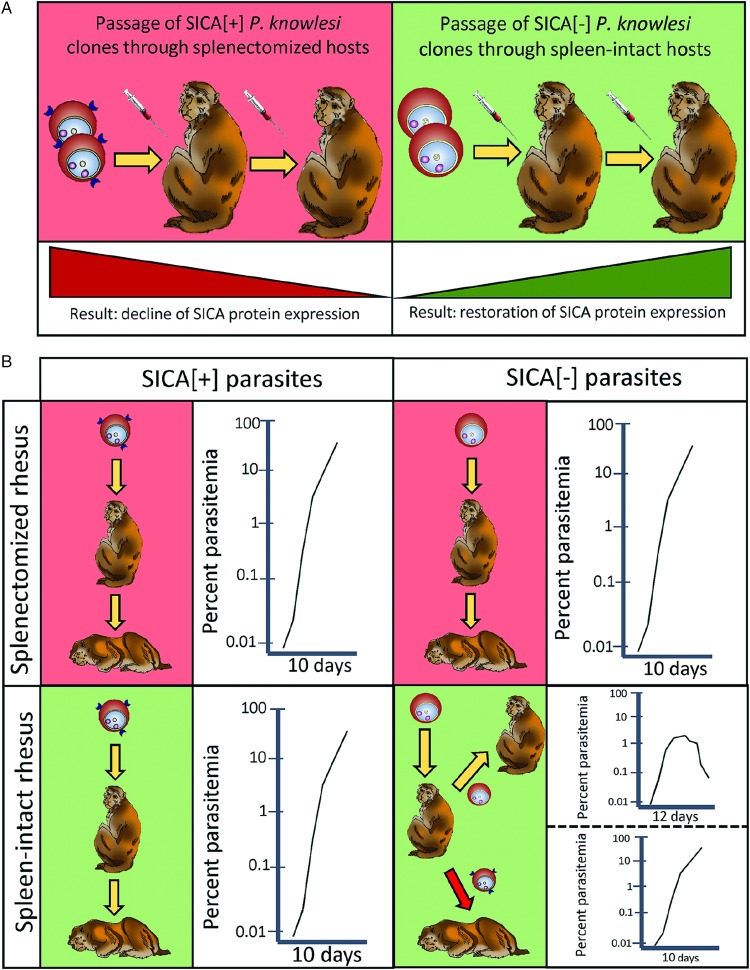
Depiction of the loss or gain of SICA protein expression in splenectomized and intact rhesus macaques (A) and of the association of virulence with the expression of SICA proteins (B), as described by Barnwell and colleagues in 1983 (Barnwell *et al.* 1983*b*). If SICA expression was regained by SICA[−] parasites in intact rhesus the parasites were highly virulent, but if SICA[−] parasites did not regain SICA expression, the infections were controlled. SICA, Schizont Infected Cell Agglutination.

The spleen also proved to be important. An ongoing mechanistic mystery is the observation that the expression of variant antigens at the surface of the iRBCs is lost during passage in splenectomized monkeys, but that expression of variant antigens can be recovered with passage in intact monkeys (Barnwell *et al.* 1982, 1983*a*, *b*). Specifically, immunofluorescence assays (IFAs) and labelling experiments by Barnwell *et al.* showed that the loss occurred in a matter of a few 24-h cycles and confirmed that the expression of SICA was associated with host factors and parasite virulence (Fig. 2). Intact rhesus monkeys almost universally succumbed to rapid, high rising parasitemias without antimalarial treatment, but they could control the virulent parasitemia if SICA proteins were not expressed. The loss of variant antigen expression in splenectomized hosts was subsequently shown to occur also with *P. falciparum* in squirrel monkeys (Hommel *et al.* 1983) and with *P. fragile* in toque monkeys (Handunnetti *et al.* 1987).

### The P. knowlesi SICAvar gene family: structure, expression, conservation and regulation

The large *SICAvar* multigene family was identified and characterized beginning in the mid-1990s, using traditional cloning, sequencing and whole genome hybridization methods (Al-Khedery *et al.* 1999). The initial report demonstrated the presence of the gene family and specifically characterized the *SICAvar* gene encoding the 205 kDa SICA protein that is expressed in the Pk1(B+)1+ clonal parasites. This gene was reported with a 10-exon structure (Al-Khedery *et al.* 1999) and later redefined as 12 exons when an approximated 12 kb intron and two additional exons were identified upstream (Lapp *et al.* 2009). An intriguing genomic rearrangement was also identified at the end of this *SICAvar* gene and shown to be associated with the *in vivo* switch from the Pk1(A+) to Pk1(B+)1+ phenotypes (Al-Khedery *et al.* 1999; Corredor *et al.* 2004; Lapp *et al.* 2009). Further research is required to determine if this observation was a unique mitotic rearrangement event or if it is a common mechanism associated with switching events. In any case, the initial 1999 report by Al-Khedery *et al.* (1999) suggested possible roles for transcriptional or post-transcriptional control of *SICAvar* expression. These early studies showed that a wide repertoire of transcripts was produced by the Pk1(B+)1+ parasites, with representation of various gene family members in a cDNA library but without particular dominant transcripts noted. Yet, northern blots demonstrated a dominant 205 kDa protein-encoding stage-specific full-length transcript in the ring stages of the Pk1(B+)1+ but not the Pk1(A+) parasites (Al-Khedery *et al.* 1999). One might predict that a majority of the iRBCs in an infection express those predominant transcripts known to make the characteristic SICA proteins in a clonal population, while a minority of iRBCs may be making other *SICAvar* transcripts. As discussed further below, single cell studies will be important to clarify whether all (or most) cells produce the repertoire of *SICAvar* transcripts observed in different SICA[+] clones.

**Fig. 3.**
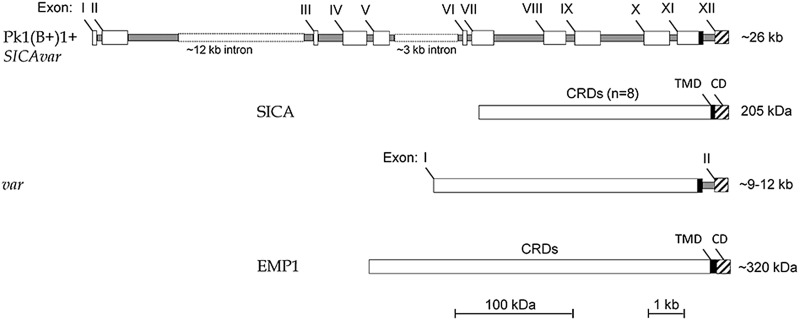
Schematic of the structure of the multi-exon *SICAvar* and *var* genes, along with the encoded proteins (SICA and EMP1, respectively). Open boxes are exons and grey rectangles represent introns; the two rectangular dotted open boxes denote particularly long intron sequences that range in size beyond the scale of the figure. CRD, Cysteine-rich domain; TMD, transmembrane domain; CD, cytoplasmic domain; SICA, Schizont Infected Cell Agglutination..

Figure 3 shows that the structure of the Pk1(B+)1+ *SICAvar* gene encoding the 205 kDa SICA protein contrasts with the much simpler two-exon structure of *var* genes *in P. falciparum* (reviewed in Galinski and Corredor, 2004). Nonetheless, both protein structures have a series of variable cysteine-rich domains (CRDs) in the large externalized portion of the protein, and EMP1 and SICA proteins were shown by proteomics to share common peptides, suggesting their evolutionary relatedness (Korir and Galinski, 2006), which is supported in a recent evolutionary analysis of *Plasmodium* variant antigen gene families and their relationships (Frech and Chen, 2013). Despite the difference in exon structures (explained as loss or gain of intronic sequences in evolutionary time), both *SICAvar* and *var* gene families possess a transmembrane domain in the penultimate exon and a final conserved exon encoding a cytoplasmic domain. It is also interesting that the 205 kDA SICA CRDs were shown to be encoded by sequence beginning at the end of each exon, interrupted by the intron sequences, and then continued at the start of the next exon (Al-Khedery *et al.* 1999).

The SICA cytoplasmic domain has been hypothesized to be functionally critical and possibly involved in signalling processes induced by specific antibodies produced against the external variant domains (Al-Khedery *et al.* 1999). This hypothesis is consistent with the concept of antibody induced switching, which was proposed beginning in 1965 to explain waves of parasitemia with different iRBC cell-surface exposed antigens (Brown and Brown, 1965), with some subsequent experimental support for this notion (Brown and Hills, 1974). As expected, *P. coatneyi*, which is phylogenetically very closely related to *P. knowlesi* (Vargas-Serrato *et al.* 2003), has a comparable large *SICAvar* multigene family (Galinski and Corredor, 2004; Chien *et al.* 2016). Additionally, while the *P. fragile* genome sequence is not yet available, antigenic variation of iRBC surface-exposed antigens has been shown for this species in the toque monkey (*Macaca sinica*) (Handunnetti *et al.* 1987), and it is likely that *P. fragile's* genome is also endowed with *SICAvar* genes. In support of this prediction, experiments with *P. fragile,* reported by Handunnetti *et al.* demonstrated a sequential pattern of variant types upon switching in the course of blood-stage infections in multiple small cohorts of toque monkeys (Handunnetti *et al.* 1987). Clearly, complementary knowledge across different primate species can inform and guide the building of mechanistic models, and include comparisons of *P. knowlesi* infections in natural monkey hosts, such as *M. fascicularis* and *M. nemestrina* (Cox-Singh *et al.* 2008).

Delving deeper into questions relating to the repertoires of *SICAvar* transcript *vs* protein expression in cloned parasites, quantitative RT-PCR and proteomics studies were performed. These studies identified the variant antigen repertoires of the Pk1(A+) and Pk1(B+)1+ parasite clones, and demonstrated a complete switch in SICA expression with dominant proteins and lesser proteins identified in each clone (Lapp *et al.* 2013). As shown for the first time in this study, the *in vivo* switch from the Pk1(A+) to the Pk1(B+)1+ phenotype resulted in the downregulation of one set of *SICAvar* genes and the upregulation of another set. The results, also reflected in northern blots, strongly suggested that both transcription and post-transcription processes were functioning to regulate the expression of these genes and proteins (Lapp *et al.* 2013).

Comparisons of SICA[+] and SICA[−] parasites furthermore revealed that the *SICAvar* gene family becomes downregulated when passaged in splenectomized animals. In the SICA[−] parasites, transcript detection is dramatically reduced, transcript signals are not detected by northern blot using specific or conserved probes and SICA products are not detected by proteomics after immunoprecipitation with an antisera that recognizes the conserved cytoplasmic domain (Lapp *et al.* 2013). These observations are consistent with possible transcriptional control mechanisms and specific post-transcriptional processing events (Galinski and Corredor, 2004); e.g. involving non-coding RNAs. The observed downregulation under these unnatural physiological conditions stresses the importance of host factors in the regulation of antigenic variation. Evidently, antigenic switch events at the genetic level that result in positive SICA expression require some unknown component or interactions provided by the presence of the spleen and specific antibodies to the expressed variant antigen, and possible other undefined regulatory factors. Of direct relevance to humans, several studies have reported the circulation of all *P. falciparum* RBC stages from splenectomized patients (Israeli *et al.* 1987; Ho *et al.* 1992; Bach *et al.* 2005; Bachmann *et al.* 2009). One of these studies specifically confirmed that the *P. falciparum var* gene family was not expressed in the iRBCs, thus connecting the lack of EMP1 expression with the loss of sequestration (Bachmann *et al.* 2009). Downregulation of the *SICAvar* family also occurs in *P. knowlesi in vitro* cultures (Lapp *et al.* 2015). Thus, long-term cultures are not particularly well suited for *SICAvar* expression studies, but *ex vivo* transfection experiments followed by *in vivo* growth can be useful to test hypotheses (van der Wel *et al.* 1997; Kocken *et al.* 2002; Kocken *et al.* 2009; Pasini *et al.* 2016). Curiously, our present culture-adapted line, derived from a line established by Kocken *et al.* (2002) did not grow when passaged back into rhesus, owl and squirrel monkeys, primate species normally highly susceptible to infection with *P. knowlesi* (unpublished data); this suggests that changes have occurred in this line over time upon *in vitro* propagation that now prohibit its successful infection and growth *in vivo*.

**Fig. 4.**
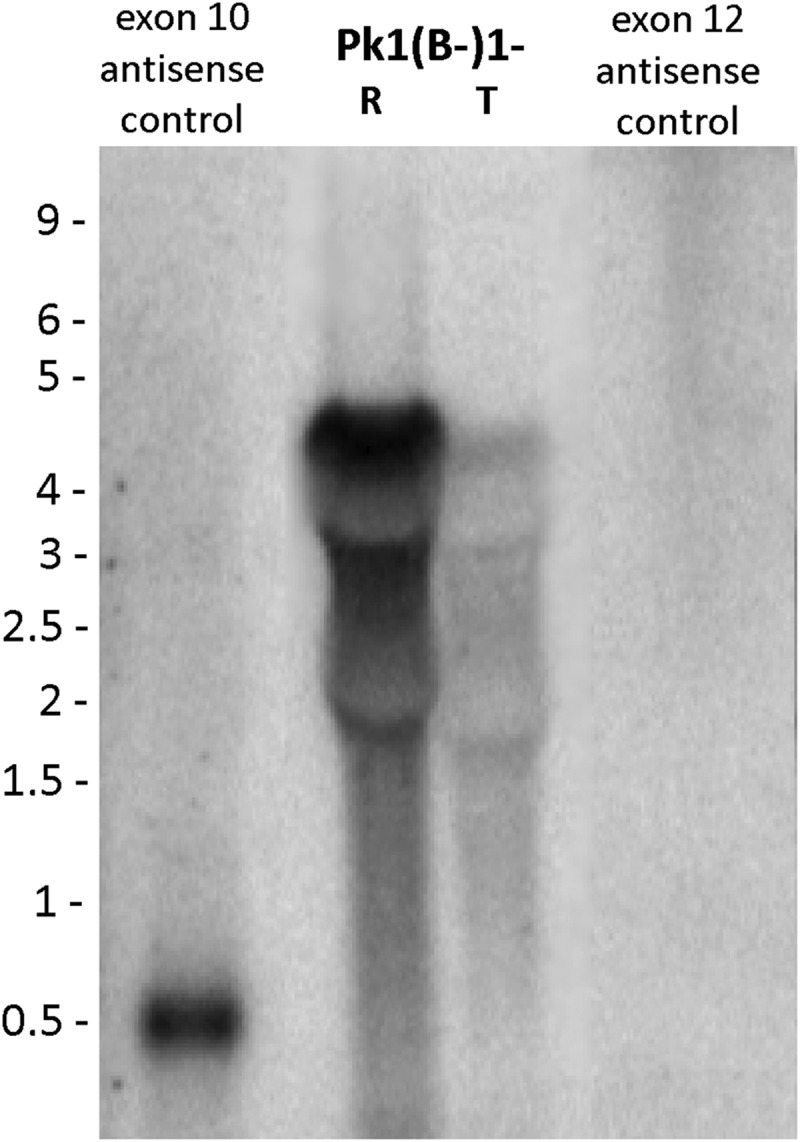
Northern blot experiment showing antisense transcripts to the *SICAvar* gene encoding the 205 kDa SICA protein are present in the SICA[-] ring (R), but not trophozoite (T) stages, of Pk1(B-)1- iRBCs. A gene-specific exon 10 sense riboprobe control hybridization, representing the probe used, is also shown. SICA, Schizont Infected Cell Agglutination.

*SICAvar* gene expression may be regulated by non-coding RNAs that are antisense to those genes, supporting the possible involvement of post-transcriptional regulatory processes (Fig. 4 and unpublished results characterizing such sequences). Observations linking non-coding RNAs with *SICAva*r gene expression, such as the presence of antisense RNAs in SICA[−] parasites that lack full-length *SICAvar* mRNA, became evident at the turn of the century, before the broad discovery of such RNAs, and their importance as regulators of gene expression in nature (reviewed in Rinn and Chang, 2012). However, a *P. knowlesi* genome sequence was not available then, which precluded further research on this topic at that time. With the first *P. knowlesi* genome sequence (Pain *et al.* 2008), however, and since with RNA-Seq transcriptome analyses (unpublished results), non-coding RNA species have become evident as predominant species. Non-coding RNA species have also been observed in *P. falciparum* and are believed to be mechanistically important for the regulation of *var* gene expression at the transcriptional level, although their precise roles are not fully understood (Amit-Avraham *et al.* 2015). Interestingly, polysome profiling analyses throughout the *P. falciparum* erythrocytic cycle identified *var* gene introns associated with the polysome fraction at the ring stage (Bunnik *et al.* 2013). These recent data indicate that control of antigenic variation and translational repression of transcribed *var* genes can occur at the translational level and involve non-coding RNA species. The field of RNA regulatory control mechanisms is burdgeoning in general, and the same is true for *Plasmodium* research reviewied in Vembar *et al.* (2016).

## THE QUEST FOR IDENTIFYING REGULATORY MECHANISMS OF ANTIGENIC VARIATION IN MALARIA

### Plasmodium falciparum, 1995 onward

**Fig. 5.**
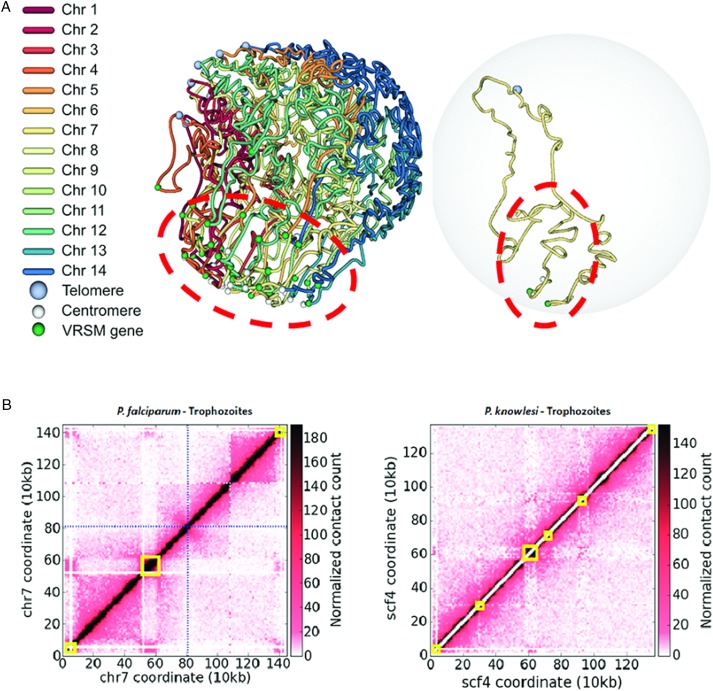
(A) 3D model of the *P. falciparum* genome – *var* genes co-localize in repressive center(s) within the red dashed ellipse (left). Co-localization of the *var* genes shapes chromosome conformation (right) in chromosome 7. (B) Hi-C contact maps of trophozoite stage parasites for *P. falciparum* chromosome 7 (left) and *P. knowlesi* scaffold 4 (Lapp *et al.* 2017). Hi-C data were generated and analysed as described (Ay *et al.* 2014), and heatmaps for normalized contact counts were created at 10 kb resolution. Yellow boxes indicate *var* and *SICAvar* gene loci for *P. falciparum* and *P. knowlesi*, respectively. *P. falciparum* gene annotations were accessed from PlasmoDB (v9·0). *SICAvar* gene annotations were curated manually (Lapp *et al.* 2017). SICA, Schizont Infected Cell Agglutination.

Numerous studies in laboratories and at field sites worldwide have been designed to better understand mechanisms regulating antigenic variation in the human malaria parasite *P. falciparum* since the discovery of the multigene *var* family in 1995 (Baruch *et al.* 1995; Smith *et al.* 1995; Su *et al.* 1995), and the publication of the first *P. falciparum* genome in 2002 (Gardner *et al.* 2002). Researchers have aimed to understand *var* gene expression patterns and switching mechanisms, and associations with immunity, illness severity and population dynamics (reviewed in Miller *et al.* 2002; Wellems *et al.* 2009; Arnot and Jensen, 2011; Guizetti and Scherf, 2013; Cortes and Deitsch, 2017). *Plasmodium falciparum* EMP1 is associated with pathogenicity mediated through the adherence of infected RBCs *via* CRDs to the endothelium of post-capillary venules and accumulated immunity to antigenic variants has been associated with the reduction of clinical severity in adults and older children (Smith *et al.* 1995; Bull *et al.* 1998; Smith *et al.* 2000; Warimwe *et al.* 2013). The ensuing body of epigenetic research has revealed processes involving histone modifications, subnuclear localization and movement of *var* genes, promoter/promoter interactions, and roles for long non-coding RNAs (Chen *et al.* 1998; Scherf *et al.* 1998; Deitsch *et al.* 2001; Duraisingh *et al.* 2005; Freitas-Junior *et al.* 2005; Chookajorn *et al.* 2007; Dzikowski *et al.* 2007; Frank *et al.* 2007; Dzikowski and Deitsch, 2008; Volz *et al.* 2012; Jiang *et al.* 2013). Silent *var* genes have been shown to co-localize with each other near the nuclear periphery, in heterochromatin regions, while a single active *var* gene was relocated elsewhere (Freitas-Junior *et al.* 2005; Lopez-Rubio *et al.* 2007; Lopez-Rubio *et al.* 2009). Recently, the development of next-generation sequencing combined with molecular assays that measure proximities of DNA (e.g., 4C-Seq and Hi-C, referring to one-*vs*-all and all-*vs*-all high-throughput chromosome conformation capture sequencing, respectively (Lieberman-Aiden *et al.* 2009; van de Werken *et al.* 2012) and histone modifications (e.g. ChIP-Seq; chromatin immunoprecipitation-sequencing) demonstrated that the *var* gene family adds a striking complexity to the genome organization and clusters of *var* genes act as structural elements that shape the genome architecture (Fig. 5A) (Lemieux *et al.* 2013; Ay *et al.* 2014; Ay *et al.* 2015); similar structural elements were also observed with *SICAvar* genes in *P. knowlesi* (Fig. 5B). The identification of specific players on the stage of complex interactions, such as PfSETvs-dependent H3K36me3 in *var* gene silencing, raised the prospect that the knowledge gained from these studies may lead to the development of translational, preventative or therapeutic tools (Jiang *et al.* 2013).

### Single cell expression

**Fig. 6.**
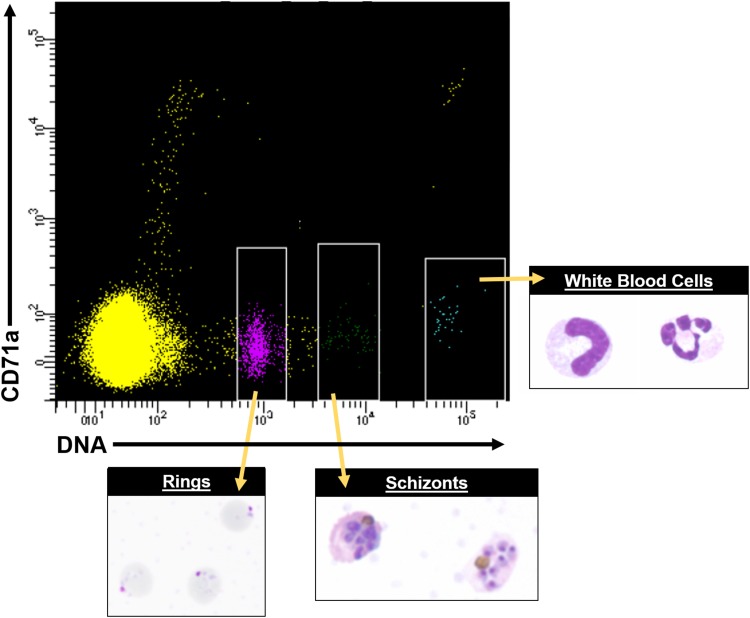
FACS data showing distinct cell populations, including different parasite stages from *P. knowlesi* infected blood, using antibodies conjugated to fluorophores and Hoechst 33342 DNA dye. A representative FACS plot from multiple experiments is shown. White rimmed boxes indicate specific cell populations that were sorted based on the single-cell expression of CD71a and DNA content, demonstrating the ability to resolve infected RBCs from white blood cells and uninfected RBCs. A representative image of each sorted population is provided. FACS, fluorescent activated cell sorting.

Most mechanistic studies on variant antigens have been performed on populations of cells, due to the historical limitations of single-cell technologies, and these populations were often derived with receptor binding/panning selection methods (Biggs *et al.* 1992; Roberts *et al.* 1992). However, recent advances in single-cell sorting and single-cell transcriptomics suggest powerful potential for studying the variant antigen gene expression profiles of single cells, or defined subpopulations, and how they relate to the larger populations from which they were derived, and possible proteomic profiles. Figure 6 shows an example of how fluorescence activated cell sorting (FACS) can be leveraged to obtain purified asexual stages from *P. knowlesi* infected blood. *In vitro* fluorescence *in situ* hybridization (FISH) and other techniques of modern molecular biology have indicated that multiple *P. falciparum var* transcripts can be present at the same time in single cells (Chen *et al.* 1998; Brolin *et al.* 2009; Joergensen *et al.* 2010), and the same has been determined for *P. knowlesi* (Lapp *et al.* unpublished data).

Single cell analyses will become important to address questions regarding the dynamics of antigenic variation *in vivo*, to clarify to what degree parasites maintain clonality with regard to *SICAvar/*SICA expression after short-term passage in naïve hosts, and how this clonality may change as a chronic infection is established. Answering such questions is key to understanding variant antigen regulation on a population level and addressing possible implications for cell–cell communication mechanisms relating to switching of variant antigen phenotypes among iRBC (reviewed in Rivkin *et al.* 2017) and possible intracellular signalling pathways as suggested in response to host antibodies (Al-Khedery *et al.* 1999). Moreover, the combination of population and single-cell transcriptomic studies along with mathematical modelling can quite readily advance understanding of mechanisms of transcriptional and post-transcriptional control, including the possible role(s) of non-coding RNAs, as on/off or antigenic switch changes are occurring over time in SICA[+] and SICA[−] infections, in intact or splenectomized animals.

## THE HOST MATTERS: THE *IN VIVO* ENVIRONMENT INFLUENCES REGULATORY PROCESSES

Over the past 10 years, evidence has been mounting that regulatory controls of the host can influence the expression of variant antigen genes, for both *P. knowlesi* and *P. falciparum*. Thus, according to our current understanding, the basic mechanisms governing antigenic variation must be evaluated from *in vivo*-derived data to capture a complete picture and the dynamics that include such influences. This point has been stressed by many researchers, taking the spleen and immune responses into account (Galinski and Corredor, 2004; Daily *et al.* 2005; Bachmann *et al.* 2009; Arnot and Jensen, 2011; Bachmann *et al.* 2011; Lapp *et al.* 2015), and recent studies involving humans and mice have shown a ‘reset’ of the expression of the variant antigen gene repertoire after passage through the mosquito vector (Lavstsen *et al.* 2005; Wang *et al.* 2009; Spence *et al.* 2013; Bachmann *et al.* 2016). However, investigations with humans or rodent models have caveats. First, longitudinal infection discovery is limited in human investigations due to ethical restrictions and the need of immediate blood-stage treatment. Second, genetic counterparts to the *var* gene family do not exist in rodent malaria species. Consequently, a deeper understanding of the mechanisms regulating antigenic variation of the *var* or *SICAvar* families – before, during and after mosquito passage – will enormously benefit from *in vivo* investigations using appropriate animal models, and there is little doubt that the host–parasite interactions at the molecular and immunobiological levels in humans will be most similar to those in NHPs.

So far, an NHP model has not been available to conduct comparable research with *P. falciparum* akin to what is possible with *P. knowlesi* or other simian malaria parasites in macaques, which is due to various factors including the limited number of *P. falciparum* isolates adapted to New World monkey hosts and the limitations of those parasite–host combinations (reviewed in Galinski and Barnwell, 2012). Work on antigenic variation of *P. falciparum* in NHPs to date has been limited to the Falciparum Uganda Palo Alto (FUP) strain and *Saimiri sciureus* monkeys (Hommel *et al.* 1983). The blood stages of this strain have been adapted to produce robust infections in these animals, but transmission through a mosquito vector has not been possible. Moreover, the animals weigh only about 1 kg, which imposes severe limitations on experimental blood volume draws in comparison with macaques. Fortuitously, as shown in Table 1, biological features of *P. falciparum* EMP1 and *P. knowlesi* SICA are quite similar, and one might legitimately expect the regulatory mechanisms to reflect corresponding similarities.

**Table 1.** Features shared by *P. knowlesi* SICA and *P. falciparum* EMP1

Variant antigen characteristic	SICA protein	PfEMP1
Large parasite-encoded proteins	✓	✓
Exposed at infected RBC surface	✓	✓
Extractable only by ionic detergents	✓	✓
Undergo antigenic variation	✓	✓
Associated with virulence	✓	✓
Encoded by large multigene family	✓	✓
Contain cysteine-rich domains	✓	✓
Subject to much diversity	✓	✓
Genes on all chromosomes	✓	✓
*In situ* transcription	✓	✓
Abundant transcription in ring stage	✓	✓
Translation in trophozoite stage	✓	✓
Antisense long non-coding RNAs in gene regulation	✓	✓
Epigenetic gene control	✓	✓
Role for spleen in expression control	✓	✓
Associated with typical cytoadherence/sequestration	×^a^	✓

a *P. knowlesi* exhibits some sequestration characteristics, which may be attributed to the expression of specific variant antigens (Miller *et al.* 1971). Adapted from Korir and Galinski, 2006 (Korir and Galinski, 2006).

### Comparative studies in macaques

Rhesus monkeys have been used for decades as well-established experimental hosts of *P. knowlesi*, however, they are not known to be infected with this species in the wild. *Macaca fascicularis* and *M. nemestrina* are natural hosts and disease reservoirs for human infections in South East Asia (Cox-Singh *et al.* 2008; Divis *et al.* 2015). These species have been studied to a lesser degree with *Plasmodium* infections, but should be utilized as well to develop within-host knowledge of antigenic variation. In contrast to rhesus, these species do not exhibit overwhelming parasitemia with *P. knowlesi* infections, nor a comparable high risk of mortality (Knowles and Das Gupta, 1932; Anderios *et al.* 2010). Clinical manifestations of *P. knowlesi* in these species more closely parallel human cases (Daneshvar *et al.* 2009), although lower parasitemias and controlled infections make them less suitable for studying and modeling switch events in longitudinal infections. Ultimately understanding the differences in the dynamics of SICA protein expression in these distinct host species may be enlightening with regard to the dramatic differences in virulence, the immune reponses and their clinical manifestations.

Aside from having a clear role in parasite virulence, the biological function of SICA proteins and their precise localizations on the host cell membrane are still unknown. As both SICA[−] and SICA[+] expand rapidly in splenectomized hosts, the function of SICA proteins is not thought to be metabolic (Barnwell *et al.* 1983*a*). *Plasmodium knowlesi* exhibits sequestration behaviour, which however is limited compared with *P. falciparum, P. coatneyi or P. fragile,* for which only the ring-stage forms and gametocytes typically circulate. In synchronous *P. knowlesi* infections, fewer than the expected number of schizonts are often observed on a morning blood smear compared with the number of ring-stage parasites observed the afternoon prior. Miller *et al*. described extensive *P. knowlesi* schizogony in several tissues in rhesus monkeys (Miller *et al.* 1971), and more recent studies showed *P. knowlesi* iRBC distribution in baboon tissues (Ozwara *et al.* 2003; Onditi *et al.* 2015). Additionally, unlike *P. falciparum* and *P. coatneyi*, the *P. knowlesi* variant antigens are not concentrated at parasite-induced protrusions at the RBC surface (reviewed in Galinski and Corredor, 2004). The much-simplified *P. knowlesi* infected RBC surface, without knobby protrusions also introduces questions regarding the minimal requirements of parasitism of the primate species and the fundamental biological function of the variant antigens across the species. *Plasmodium knowlesi* manages with a shorter asexual blood-stage life cycle of ~24 h, *vs* ~48, and simply by comparison with producing a number of sparse internal caveolae structures at the iRBC surface. The function(s) of these structures has yet to be defined, though they are of high interest, as they likely perform critical physiological functions, and could become targets of intervention against *P. knowlesi,* as well as other species.

## SYSTEMS BIOLOGY: THE WAY FORWARD

Enormous advances in modern biology, including the omics revolution, and in mathematical and computational modeling are aligning and current research suggests that methods of systems biology have great potential for elucidating various aspects of antigenic variation in *P. knowlesi* during experimental infections of NHPs. For instance, the combination of targeted experimental and modelling methods could focus on the parasite's growth and development *ex vivo*, as well as in both NHP and mosquito hosts, and ultimately reveal molecular mechanisms that govern antigenic variation *in vivo* and by definition contribute to successful parasitism, transmission and pathology. This emerging holistic research strategy will demand novel mathematical models and computational approaches, customized for the *P. knowlesi* rhesus-macaque model systems, to characterize gene and protein expression of variant antigens, and immune responses, as well as the principles of the switching dynamics and epigenetic players.

Through the integration of multiple data sources, careful interpretation of epigenetic changes, and the identification of external stimuli that activate these changes, novel signalling pathways may be discovered that might become the target of future vaccine or antimalarial intervention strategies. An improved understanding of the multilayer regulation of the *SICAvar*/*var* gene expression including epigenetics is within reach, and such knowledge will lead to a greater understanding of parasite immune evasion mechanism and host survival.

Antigenic variation is such a complex process that one can easily imagine diverse modeling strategies toward investigating different aspects. These could mathematically describe anything from the molecular interactions of SICA proteins with other intracellular proteins, to the sequence of expressed genes, as well as the overall competitive advantage that different *SICAvar* genes might confer to clonal populations. Here we briefly sketch two such strategies.

### Stochastic modelling of switching patterns

Several mathematical models have been proposed to describe and predict mechanistic features of antigenic variation in a number of pathogens, including *Plasmodium* (Recker *et al.* 2004; Blyuss and Gupta, 2009; Recker *et al.* 2011; Buckee and Recker, 2012; Noble and Recker, 2012; Severins *et al.* 2012), *Streptococcus*, *Neisseria* (Lipsitch and O'Hagan, 2007), trypanosomes (Agur *et al.* 1989; Antia *et al.* 1996), and HIV (Nowak and May, 1991). The main targets of these studies have been switching patterns, infection duration and chronicity. As an example, switching patterns of *var* gene repertoires were modelled based on *in vitro* derived data from *P. falciparum* blood-stage cultures. In one of these studies, Recker's group observed a non-random, highly structured switching pathway where an initially dominant transcript switched *via* a set of intermediates either to a new dominant transcript, or back to the original (Noble *et al.* 2013). It was furthermore proposed that *var* genes have intrinsic probabilities of being activated or silenced and that switching patterns can be inferred from transcription time courses of several parasite populations from the same isolate, each starting with different variant distributions (Noble and Recker, 2012).

As an alternative to this strategy, and capitalizing on *P. knowlesi* model systems, it is in principle possible to design and test stochastic models that permit a dynamic characterization of switching patterns *in vivo*. For instance, one could develop Markov chain models to shed light on antigen switching during longitudinal infection of NHPs. Such models contain states, which here are combinations of expressed *SICAvar* genes, epigenomic profiles, or protein-based phenotypes and transitions, which correspond to switches among states and can be estimated from RNA-Seq studies of expressed genes before and after switches. Interestingly, this type of model automatically subsumes two null hypotheses as special cases, namely, that the switching is entirely deterministic, or that it is entirely random. The truth is naturally bounded by these two scenarios. Specifically, it will be of interest to study the ‘next set’ of expressed *SICAvar* genes, if some particular set is observed on different occasions. It is clear that such a strategy cannot be pursued in a human host, but it is possible to study these transitions in NHP infections. A disadvantage of the Markov approach is that it has no memory. That is, the probability of switching from one state to another is implicitly assumed to depend only on this state, but not on the history of the process before entering this state. With sufficient data, however, Markov chain models with memory can be established (Ross, 2006). If memory remains a problem, it is also possible to formulate differential equations whose state variables are the probabilities of expressing a certain gene set. This method accounts for some memory and is vaguely reminiscent of the Chemical Master Equation approach, but here leads to a homogeneous Poisson process that has an explicit solution (Stumpf *et al.* 2017). One could furthermore reformulate the process as a stochastic process with ‘short memory approximation,’ expressed as a stochastic differential equation (Zhabin, 2004; Goldwyn *et al.* 2011).

### Kinetic modeling of infections

Rather than attempting to predict which variant might be expressed next, given a certain present state, one could ask if the expression of certain variants confers a dynamic advantage over other alternatives. Such an advantage could be due to an intrinsic growth advantage, enhanced immune evasion, or suppression of other variants. So far, there have been only a few attempts to model the kinetics of malarial antigenic variation and its impact on the outcome of the infection (Childs and Buckee, 2015). For example, Childs and Buckee analysed *P. falciparum* infections but suggested that antigenic variation does not explain the length of chronic infections (Childs and Buckee, 2015). Antia's group (Johnson *et al.* 2012) proposed that persistent infections could be explained by a high level of immunodominance coupled with either killing saturation or immune exhaustion. They also suggested that immune exhaustion plays an important role in the determination of when the primary infection should be treated in order to allow development of protection against a secondary infection.

As an alternative to these documented approaches, and with the aim of characterizing the immunogenicity, growth rates and competition among different variants, one could pursue a combination of an SIR (Susceptibile, Infected, Recovered) type of model with a Lotka-Volterra model of interspecies competition. The SIR formulation could be used to describe how different variants infect susceptible RBCs and thus produce different SICA-expressing iRBCs. The Lotka-Volterra formulation could add a variant-specific and cross-reactive (Johnson *et al.* 2012) immune response that could control the growth of each variant, and a set of interaction terms between parasite variants would characterize the suppression or promotion of one variant over another. This approach could just as well be applied to coinfections with different strains or species.

###  Key experimental considerations

As a first step towards these goals, a high-quality *P. knowlesi* nuclear genome assembly with manual annotation was generated recently and called the ‘MaHPIC Pk Genome Sequence’ (see Lapp *et al.* 2017 in this Special Issue). This nuclear genome sequence can serve as the basis for studies of gene regulation as a whole, for capturing regulatory gene networks, and specifically for propelling the field forward toward understanding *SICAvar* expression and switching dynamics *in vivo*. The combination of long reads Pacific Biosciences (PacBio) with Hi-C technology (Kaplan and Dekker, 2013), which generates genomic distance proxies to accurately position contigs without requiring sequence overlap, has greatly improved *de novo* scaffolds in *P. knowlesi;* this is contrasted in Table 2 with the basic information on the original nuclear genome sequence (Pain *et al.* 2008). Similar to the original genome sequence, the MaHPIC Pk Genome Sequence is based on genomic DNA from the Pk1(A+) clone of the Malayan strain of *P. knowlesi*. The 2008 assembly provided a glimpse into the *SICAvar* family on all 14 chromosomes, but had 190 gaps, many *SICAvar* fragments and misplaced genes. In total 29 full-length *SICAvar* genes were identified in 2008, and we have now confirmed at least 136 *SICAvar* genes (117 Type I and 19 Type II) and 22 ‘*SICAvar* gene segments’ that mainly (and curiously, as they may in fact have a functional purpose) contain the first two or last 3 exons (Lapp *et al.* 2017). The current MaHPIC assembly is advanced in terms of the overall confirmed 24·6 M genome size, with high sequencing coverage (151X with PacBio and 68X with Hi-C), 15 contigs and only 25 gaps (Table 2). Another *P. knowlesi de novo* genome assembly, based on the use of PacBio and culture adapted lines, was also reported recently (Moon *et al.* 2016): a thorough manual assessment of the *SICAvar* sequences in this assembly would be of interest as performed for the MaHPIC Pk Genome Sequence (Lapp *et al.* 2017).

**Table 2.** MaHPIC Pk Nuclear Genome Sequence: basic information comparisons with previously reported *P. knowlesi* nuclear genome sequences from the Malayan Strain and two *in vitro* culture adapted lines

Malayan strain^a^	Technology	Size	CDS	Coverage	Chr/ scaffolds	Contigs	Gaps
PKNOH/MaHPIC (Lapp *et al.* 2017)	PacBio+HiC	24·8 M	5217	151X (PacBio) 68X (Hi-C)	14	14	25
PKNH/Sanger v2 PlasmoDB^b^ 2017	Illumina	24·4 M	5282	UA	14	148	77
PKNH/Sanger v1 (Pain *et al.* 2008)	WGS 4 & 40 kb insert libraries	24·4 M	5188	8X	NA	204	190
*In vitro* culture adapted lines							
PKNA1-C.2 (Moon *et al.* 2016)	PacBio	24·4 M	5172	121X	14	45	NA
PKNA1-H.1 (Moon *et al.* 2016)	PacBio	24·0 M	5138	126X	14	37	NA

CDS, coding sequences; Chr, chromosomes; WGS, whole genome sequencing; NA, not applicable; UA, unavailable.

a Pk1(A+) clone (Howard *et al.* 1983).

b PlasmoDB, release 32, April 20, 2017 (Aurrecoechea *et al.* 2009).

To advance the field of systems biology of antigenic variation processes and mechanisms, *P. knowlesi –* macaque longitudinal infections can be monitored daily and and blood samples collected strategically with the goal of capturing and defining molecular host-parasite events and immune reponses related to the switching dynamics. Using methods reported recently for *P. coatneyi* and *P. cynomolgi* (Fonseca *et al.* 2016; Joyner *et al.* 2016), time series data can be generated for clinical parameters and all cell types available in blood count measurements and integrated with omics information to build and calibrate cellular models of antigenic variation. Such studies can be performed in spleen-intact and splenectomized macaque hosts. Chromatin remodelling and histone modifications are dynamic epigenetic changes responsive to external stimuli and, hence, can be integrated into these dynamic models, at least in principle.

### Concluding remarks/future directions

Many hypotheses regarding the molecular and immunobiological basis of antigenic variation in malaria, can effectively be addressed with *in vivo* NHP infection models, beyond what is possible with humans, and advance quickly with the use of modern methods of multi-omic data generation and analysis, and sophisticated strategies of integrating heterogeneous information with tools from systems biology. In the past 20 years, many breakthroughs in our understanding of variant antigen gene expression have involved *P. falciparum* and *in vitro* culture systems, but it has become abundantly evident that gene and protein expression patterns are influenced by host factors. This truth is problematic for fully understanding antigenic variation in humans, because clinical studies with humans require treatment of blood-stage parasitemias. The well-established *in vivo* NHP – *P. knowlesi* infection model system provides a superb alternative for investigating longitudinal infections. With this host-pathogen system, the dynamics of antigenic variation and switching processes can be explored, and the full lifecyle interplay can be studied between the primate and vector hosts, including epigenetic regulation, cell–cell communication mechanisms and uncharted aspects of host-factor involvement; including the elusive role of the spleen. The potential of mechanistic studies is now opportune with the release of the MaHPIC Pk Genome Sequence, with the correct placement and annotation of 136 members of the *SICAvar* gene family (Lapp *et al.* 2017). Researchers can now test whether and how (i) *Plasmodium* variant antigen expression and switching patterns undergo predictable dynamics in the primate and mosquito hosts; (ii) epigenetic mechanisms within the parasite can drive *in vivo* switching of variant antigen expression; (iii) host factors can drive epigenetic control of antigenic variation in the parasite; and (iv) variant antigen switches and the switching rate correlate with antibody response patterns. As the scientific community takes on these challenges, it may also become viable to effectively explore selected question using *P. falciparum* – NHP models. One day, interventions may come to light that can interrupt switching mechanisms and thus accelerate the elimination of parasites, which would otherwise remain disguised and survive the host's immune response.
